# Alterations in the Platelet Transcriptome Mediate Prenatal Thirdhand Smoke Exposure Associated Thrombogenicity via Integrated miRNA-mRNA Regulatory Networks

**DOI:** 10.3390/ijms26157633

**Published:** 2025-08-07

**Authors:** Hamdy E. A. Ali, Ahmed B. Alarabi, Fatima Z. Alshbool, Fadi T. Khasawneh

**Affiliations:** 1 Department of Pharmaceutical Sciences, Irma Lerma Rangel College of Pharmacy, Texas A&M University, Kingsville, TX 78363, USA; haali@tamu.edu (H.E.A.A.); alarabi@tamu.edu (A.B.A.); 2 Department of Pharmacy Practice, Irma Lerma Rangel College of Pharmacy, Texas A&M University, Kingsville, TX 78363, USA; falshbool@tamu.edu

**Keywords:** platelets, transcriptome, exposure, integrated analysis, thrombosis

## Abstract

Cigarette smoking is acknowledged as the most preventable risk factor for thrombogenesis-associated cardiovascular disease. Mice prenatally exposed to the thirdhand smoke (THS) form of tobacco exhibited a higher tendency to develop occlusive thrombosis, along with enhancement of several platelet functional responses. Our objective was to investigate whether prenatal (in utero) THS exposure impacts the platelet transcriptome, resulting in enhanced platelet functional responses, thereby underlying THS-associated thrombogenicity. Blood samples obtained from twenty male mice prenatally exposed to THS, along with an equal number of age-matched male mice exposed to clean air (CA) as a control, were divided into pools of five animals and used to prepare leukocyte and red blood cell-depleted platelets. RNA sequencing for mRNA and microRNA (miRNA) was utilized to analyze and compare the platelet expression profiles of the two exposure groups. RNA seq analyses revealed distinct changes in both gene expression and miRNA profiles, with 448 coding genes and 18 miRNAs significantly altered between the two groups. miRNA–mRNA interaction analysis highlighted 14 differentially expressed miRNAs that potentially target 120 of the differentially expressed genes in our data set. Interestingly, altered genes in miRNA–mRNA pairs were functionally enriched into pathways associated with platelet physiology, including platelet activation, signaling and aggregation, and cellular response to chemical stimuli. Our findings establish—for the first time—that prenatal exposure to THS modifies the platelet transcriptome, thereby rendering platelets hypersensitive to stimuli and more prone to thrombogenicity. Additionally, we illuminate the coordinated function of platelet miRNA and mRNA targets in mediating this response.

## 1. Introduction

Thirdhand smoke (THS) denotes the enduring toxic residues of secondhand smoke (SHS) that end up depositing on surfaces and objects in environments where tobacco has been used, such as homes [1,2,3,4]. THS remains in residential environments for extended periods, often lasting weeks to months after the last smoking [5,6,7,8,9,10]. Additionally, the toxicants present in THS undergo chemical transformations over time, potentially increasing their toxicity [1,2,3,11]. Routes of exposure to THS include skin absorption, inhalation, and ingestion. Given that the routes of exposure to THS are primarily skin absorption, inhalation, and ingestion [12,13,14], it is considered/thought to be more toxic due to its capacity to produce more toxicants in the blood of the exposed person [15,16].

Evidence has highlighted THS as a significant threat to human health [1,9,17,18,19], especially among vulnerable populations such as infants and children, as well as adults and workers in smoking-permissive environments [16]. THS has been demonstrated to elevate cancer risk, as its toxic profile reveals an accumulation of carcinogens, including nitrosamines and potent lung carcinogen NNK, on surfaces within smokers’ homes [20,21]. THS has been demonstrated to exert a genotoxic effect on human cells by increasing DNA strand breaks. Also, THS exposure has been demonstrated to elevate the risk of cardiovascular disease [22,23,24,25]. Furthermore, utilizing animal and human exposure models, THS exposure was linked to negative health outcomes, including perturbed blood cell counts and oxidative stress in skeletal muscles of exposed mice [26]. Additionally, we previously documented that THS increases the risk of thrombogenesis [22,27]. The adverse effects of THS exposure, such as dysregulated metabolic pathways, amongst others, have also been reported in several in vitro studies [28,29].

Tobacco smoke exposure is also known to be associated with alterations in the transcriptomic profile in almost all tissues, regardless of whether the exposure takes place directly or indirectly [30]. The oral mucosa [31] and lungs [32] exhibit alterations in their expression profiles in response to direct exposure to cigarette smoke, whereas indirect exposure affects the blood transcriptome [33]. Negative health effects on the transcriptome have also been demonstrated in vitro in human airway epithelial cells [34] and rat tracheal explants [35]. THS has also been associated with transcriptomic and proteomic alterations in stem cells [36] and plasma [26], respectively.

In terms of the effects of prenatal (in utero) exposure, human studies have reported that maternal exposure is associated with adverse birth outcomes, including reduced gestational period [37] and impaired lung function and immune system development [38,39], amongst others [40]. Moreover, a plethora of reports have linked maternal smoking and adverse health outcomes with epigenetic alterations in the offspring. Indeed, tobacco smoke has been reported as a strong modulator of DNA methylation [41]. A genome-wide methylation meta-analysis identified about 6000 differentially methylated CpGs in the cord blood of children related to maternal smoking [42]. Similar effects have also been reported in other tissues, including the placenta [43] and fetal lungs [44], amongst others [45]. Interestingly, some of the smoke-induced changes in DNA methylation in maternally exposed children were found to be maintained after birth, through infancy and even up to adulthood [46]. Maternal smoking-associated DNA methylation patterns have also been correlated with changes in transcriptional activity when occurring in regulatory elements of nearby coding genes [33]. However, few reports have associated the effects of in utero cigarette smoke exposure directly with fetal transcriptomics.

We recently provided evidence that prenatal exposure of mice to THS renders platelets of their offspring hyperactive at 10–12 weeks of age, resulting in a prothrombotic phenotype [25]. However, the mechanism underlying this phenotype at the molecular level remains to be investigated. To this end, despite the absence of the nucleus, platelets contain a vast array of RNA molecules that encompass all RNA species and are enriched with RNA representing all functional classifications of nucleated cells, including mRNAs and regulatory RNAs (e.g., miRNA) [47]. In fact, platelets are able to perform de novo splicing and translation upon activation [48]. Given these capabilities, the platelet transcriptome is constantly changing in response to various effectors, such as environmental stressors [49]. Based on the aforementioned considerations, we hypothesized that prenatal THS exposure modifies the platelet transcriptome in the offspring in a manner that promotes a prothrombotic state. We employed whole-transcriptome sequencing (mRNA and miRNA) to characterize gene expression alterations associated with prenatal THS exposure and performed an integrated analysis for both differentially expressed mRNA and miRNA to understand how variations in the expression of miRNA might affect diverse networks of gene expression, as well as their functional consequences for platelet signaling.

## 2. Results

### 2.1. Experimental Design

**Figure 1:**
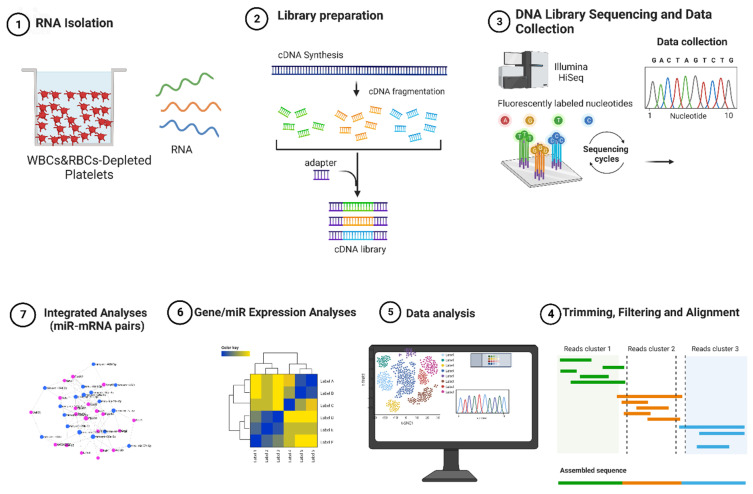
Overview of the experimental design. Total RNA isolated from White Blood Cells- (WBC)- and Red Blood Cells- (RBC)-depleted platelets; the cDNA libraries were generated for both mRNA and small RNA, and the sequenced reads were mapped against a reference transcriptome. Differentially expressed mRNA/miRNAs were determined and prepared for the downstream analyses, including functional enrichment and integrated analysis.

We hypothesize that in utero THS exposure modifies the platelet transcriptome in a manner that explains the prothrombotic state observed in the prenatally THS-exposed mice. To address this notion, RNA sequencing was conducted to evaluate and compare the gene expression profile and miRNA profile of platelets of the prenatal/in utero THS- and Clean Air- (CA)-exposed mice (Figure 1). We aimed to identify the effects of alterations in both profiles triggered by THS exposure to functional enrichment analysis on the differently altered coding target genes of the altered miRNAs to determine whether they are enriched in functional pathways critical to platelet physiology in the offspring. Given that platelets are anucleate, transcriptional control over mRNA expression is inert, suggesting a potential role for post-transcriptional regulation via platelet miRNAs. Therefore, we employed integrated miRNA–mRNA analysis to better understand how miRNA and mRNA work together to regulate platelet signaling following prenatal THS exposure and identify the mRNA–miRNA interactions relevant to alterations in platelet signaling due to THS exposure.

### 2.2. Prenatal THS Exposure Triggers Changes in Gene Expression in the Platelets of the Offspring

**Figure 2:**
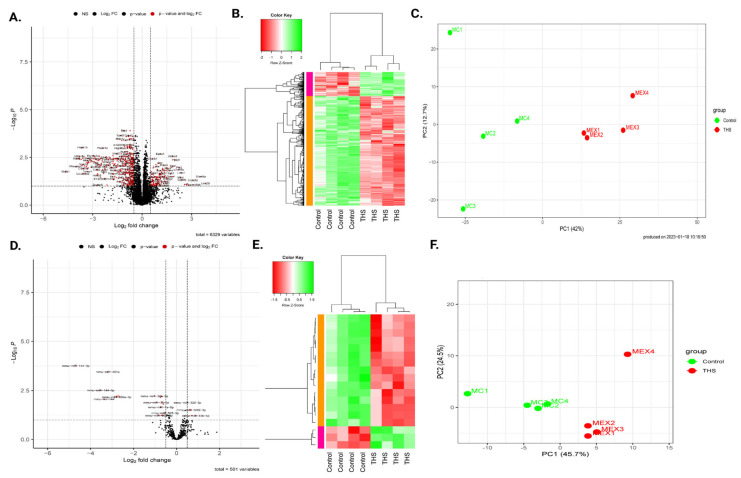
Expression landscape for platelet mRNA and miRNA profiles for the prenatal Third Hand Smoke (THS) and Clean Air (CA)/control exposure groups. (**A**,**D**) Volcano plot comparing mRNA (**A**) and miRNA (**D**) expression profiles for the THS-exposed group vs. the CA-exposed group. The horizontal line corresponds to a log2-fold change, while the vertical line indicates a log10 *p*-value. The dots are colored red if classified as significantly up-, or down-regulated. (**B**,**E**) Heat map of the hierarchical clustering of gene expression of mRNA (**B**) and miRNA (**E**). The dendrogram on top shows the clustering of the samples (clustered by exposure), and the dendrogram on the side shows the clustering of mRNA/miRNA (clustered for expression). The colors in the heat map represent RNA expression intensities, scaled from red (lowest expression) to green (highest expression). (**C**,**F**) Principal Component Analysis (PCA) of sample expression profiles in the two exposure groups for mRNA (**C**) and miRNA (**F**). PCA was performed using normalized RNA-Seq data, and for both groups, the samples are separated by exposure and expression.

The gene expression pattern of the in utero THS-exposed platelets and their CA-exposed counterparts was visualized using Principal Component Analysis (PCA) to explore the transcriptome-wide trends and discriminate differences (Figure 2C). Next, we generated a volcano plot to highlight differences in gene expression (Figure 2A), which showed that the expression of 448 genes (see Supplementary Table S2) is dysregulated. Indeed, the THS-exposed platelets showed upregulation of 86 genes and downregulation of 362 genes compared to their CA-derived counterparts, using |FC| ≥ 1.5 and FDR ≤ 0.05 as the cutoff criteria. The differentially expressed genes (DEGs) were then subjected to hierarchical clustering, which revealed that prenatally THS- and CA-exposed platelets did exclusively group into two distinct clusters (Figure 2B).

### 2.3. Functional Enrichment Analysis of Differentially Expressed Genes

**Figure 3:**
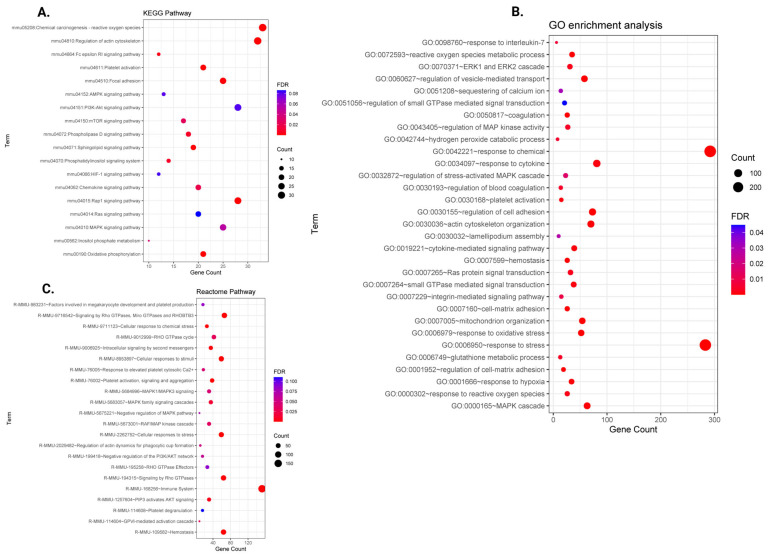
Functional enrichment analysis of pathways identified as affected by prenatal THS exposure. Bubble chart for Kyoto Encyclopedia of Genes and Genomes (KEGG), Gene Ontology (GO), and Reactome functional enrichment analyses of differentially expressed genes in platelets of prenatally THS-exposed mice. (**A**) The most enriched functional pathways according to KEGG. (**B**) The top (GO) enriched pathways (biological processes). (**C**) The most significantly enriched pathways according to Reactome.

The top DEGs in THS-exposed platelets meeting the criteria of |FC| ≥ 1.5 and FDR ≤ 0.05 were input into the Database for Annotation, Visualization and Integrated Discovery (DAVID). Using GO biological process (BP) terms, our differentially altered genes were found to be over-represented in several important signaling pathways connected with platelet function (Figure 3B and Supplementary Table S4). In fact, DEGs following THS exposure were significantly enriched into biological process GO terms that are linked to platelet physiology, which we categorized into seven main groups. These pathway categories include hemostasis and coagulation, mitochondria and oxidative stress response, and other pathways involved in platelet signal transduction (e.g., integrin-mediated signaling pathway, etc.).

In addition, DEGs showed significant enrichment into functional pathways that are crucial for platelet physiology in the KEGG database (Figure 3A and Supplementary Table S5). These pathways include platelet activation and the PI3K-Akt signaling pathway, amongst others. Furthermore, feeding the DEGs into the Reactome database also revealed several important pathways for platelet function, including hemostasis, signaling and aggregation, etc. (Table 1 and Figure 3C).

### 2.4. Prenatal THS Modifies Platelet miRNA

We compared the miRNA expression profiles of the prenatal and clean air-exposed platelets and conducted clustering analysis. Using the same criteria of |FC| ≥ 1.5 and FDR ≤ 0.05 applied to the mRNA data set, our analysis identified 18 differentially expressed miRNAs, with 3 upregulated and 15 downregulated between the two exposure groups, as illustrated in the volcano plot (Figure 2D–F and Supplementary Table S3). Moreover, hierarchical clustering and PCA revealed a clear separation in the differentially expressed miRNAs.

### 2.5. Integration of miRNA and mRNA Expression Data

To better understand how the interaction between the recognized DE mRNA and miRNA can modulate platelet signaling and potentially contribute to THS thrombogenicity, we assessed the integration of miRNA and mRNA (pairs of potential target interaction relationships). miRNA–mRNA pairs were considered to be of biological importance when a change in miRNA expression was accompanied by a significant change in mRNA expression in the opposite direction. Given that miRNA and mRNA expression profiles are obtained from the same set of samples, we identified the validated and/or predicted targets for our miRNAs and searched for coding genes that overlap with those that were previously demonstrated to be differentially expressed in our RNA sequencing data set. According to this validation, 14 of our differentially expressed miRNAs were found to potentially target 120 of the 448 DE coding genes in our sequencing data.

**Figure 4:**
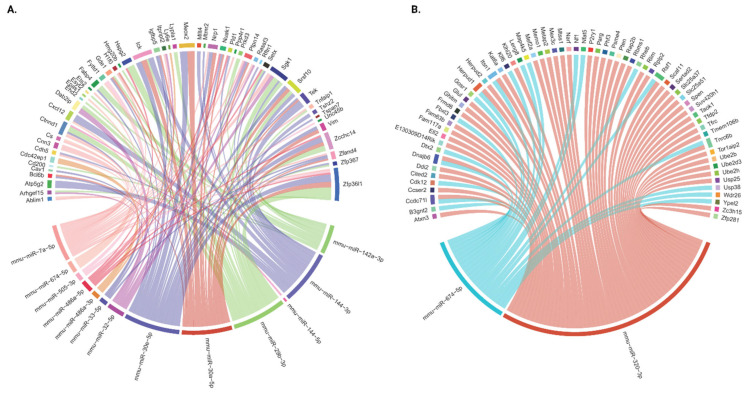
Integrated miRNA–mRNA Analysis. Circos plots displaying the interaction network of THS exposure-driven miRNA and their mRNA targets; downregulated miRNAs (**A**) and upregulated miRNAs (**B**). High-confidence miRNA cluster targets were established based on the specified criteria: (1) target genes showed differential expression with a false discovery rate (FDR) of less than 0.1 and fold change (FC) greater than 1.5 or less than −1.5; (2) the FC of mRNA and miRNA exhibited opposite directions between in THS- and CA-exposed mice using 14 databases. The databases involved in the process of integrated miRNA–mRNA analysis include miRecords, miRTarBase, TarBase, DIANA-microT, ElMMo, MicroCosm, miRanda, miRDB, PicTar, PITA, TargetScan, miR2Disease, PharmacomiR, and PhenomiR.

Thus, we constructed an interaction network between the dysregulated genes and miRNAs in response to prenatal THS exposure, as shown in Figure 4. The 120 prioritized miRNA–mRNA target pairs are presented, with their respective database-derived target predictions and/or experimental validations in Supplementary Tables S4–S6. The fact that 14 DE miRNAs interacted with 128 DE target genes indicates that miRNAs are intricately connected to their targets. As depicted in the circular plots (Figure 4), there are two clusters representing the interconnected miRNA–mRNA pairs: one in which the miRNAs are downregulated (Figure 4A; Supplementary Tables S4 and S6) and the other that represents those that are upregulated (Figure 4B; Supplementary Tables S5 and S6). Amongst the ‘downregulated miRNA cluster’ are mm-miR-7a-5p, 29b-3p/30e-5p/30a-5p, and 144-3p, which, together, target 76 DE genes. Therefore, these miRNAs represent notable examples of an elevated miRNA cluster, as each of them targets at least 15 DE genes. Furthermore, each of these genes connects with at least one other miRNA. In addition to these five big clusters within the downregulated miRNAs, there are six smaller ones, with one miRNA targeting a small number of mRNAs. These clusters include mmu-miR-142b-3p, 32-5p, 486a-3p, 486a-5p, 33-5p, 505-3p, and 144-5p, which target 10, 7, 4, 4, 3, 3, and 1 mRNA, respectively. Interestingly, these miRNAs also target shared genes; for example, Sgk1 is a shared target for mm-miR-7a-5p, 29b-3p, 32-5p, 142a-3p, and 144-3p (see Supplementary Tables S4 and S6). Interestingly, some of these genes are experimentally validated targets for more than one miRNA: for example, Ptpn14 for mm-miR-30e-5p, 30a-5p, and 7a-5p (Supplementary Tables S4 and S6).

On the other hand, upregulated miRNAs mm-miR-320-3p and 674-5p comprise two large clusters that are even “bigger” than those of the downregulated miRNA. For example, while mm-miR-320-3p is found to target 53 genes, 674-5p targets 21 genes. However, unlike the downregulated miRNA clusters, none of these targets has been experimentally validated, which warrants future investigation. Also, there is not as much overlap between these two miRNAs regarding shared targets, as observed in the downregulated clusters. In fact, only six genes have been observed as shared targets by the two upregulated miRNAs, which are Ccdc71l, Dnajb6, Herpud1, Itsn1, Rsf1, and Tnrc6b.

### 2.6. The miRNA–mRNA-Regulated Functional Pathways

As indicated before, our miRNA–mRNA integrated analysis identified 120 mRNAs that are likely targeted by DE miRNAs in the THS-exposed platelets, which, when introduced into gene enrichment pathway analysis, revealed over 40 significant platelet function-related pathways (Table S3). Most of the enriched pathways were found to have been previously recognized when the whole set of DEGs were fed into gene enrichment tools. KEGG enrichment identified pathways such as those related to PI3K-Akt signaling, focal adhesion, Wnt signaling, AMPK signaling, platelet activation, etc. Additionally, the Reactome enrichment analysis highlighted integrin cell surface interactions, post-transcriptional silencing by small RNAs, platelet activation, signaling, and aggregation, amongst others. In addition, the miRNA enrichment network highlighted pathways including PI3K-Akt signaling, mTOR signaling, Rap1 signaling, etc.

### 2.7. Q-PCR Validation of Prenatal THS-Associated Transcripts

**Figure 5:**
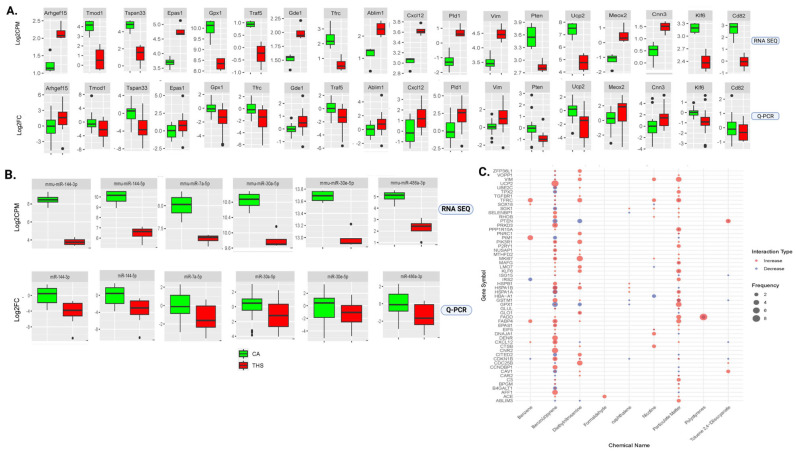
Q-PCR validation for RNA seq and THS chemical–gene interaction data. RNA was extracted from RNA isolated from THS- (n = 20) and CA-exposed (n = 20) mice of a distinct exposure cohort, mRNA and miRNA were reverse-transcribed, and RT-qPCR expression analyses were performed for the selected mRNA (**A**) and miRNAs (**B**). The amounts of transcripts for mRNA were normalized to the β-Actin (ACTB), β2-microglobulin (B2M), and Ribosomal Protein Lateral talk subunit P0 (RPLP0), whereas miRNAs were normalized to RNU1A1 and 5S rRNA. The relative mRNA expression is plotted as fold changes calculated by the 2^−∆∆Ct^ method. (**C**) Interaction between differentially expressed genes in the RNA seq data set with the top 10 chemicals of the THS toxic profile as predicted by the toxicogenomic database.

Next, in a manner that covers a wide range of fold variations rather than being solely based on their FC rank (Figure 5, Table 2 and Table 3), we selected 18 genes and 9 miRNAs from our RNA data set—including both up- and downregulated transcripts—for qRT-PCR validation. It is important to note that the validation was carried out using a different cohort of prenatally THS-exposed mice. Three genes were found to have borderline significant *p* values, whereas another gene followed the same trend as RNA seq without achieving significance and a separate one could not be identified by Q-PCR according to the qRT-PCR results of the coding genes, which corroborated the substantial down- or upregulation of 15 genes. With the exception of two miRNAs that maintained the trend with borderline significance and one miRNA that could not be detected by Q-PCR, the ten miRNAs chosen for Q-PCR validation also replicated the trend exhibited by the RNA seq data set.

### 2.8. Gene–Chemical Component Enrichment Using the Comparative Toxicogenomic Database

We conducted an integrated toxicogenomic enrichment using the Comparative Toxicogenomic Database (CTD) to predict the toxic chemical components of THS that might play roles in disrupting the platelet transcriptome (mRNA). Toxicogenomic enrichment may provide evidence for the potential interaction of chemical components of the toxic profile of THS with the altered gene expression in our data set that may have driven the thrombogenicity observed in the offspring of exposed females. Indeed, the altered expression of these genes is most likely attributed to interaction with one or more chemicals in the toxic profile of THS. Importantly, our analysis showed that the main THS chemicals exhibit interactions with most of the genes with an altered expression in the THS-exposed mice (Figure 5C), although we focused on the top ten. Indeed, benzo(a)pyrenes showed interactions with about 350 of the 448 differentially expressed genes, followed by particulate matter, which interacted with more than 150 genes; then came diethylnitrosamine and nicotine. Importantly, Ucp2 [50], Rhob [51], P2ry1 [52], Pten [53], Cav1 [54], and Cxcl12 [55], which are known to modulate platelet function, were among genes that showed high levels of interaction with one or more of the top 10 components of the toxic profile of THS.

## 3. Discussion

Platelets possess a dynamic transcriptome that is very responsive to external stimuli, with the platelets’ miRNA and mRNA profiles reflecting their functional diversity in mice and humans [56]. On this basis, we investigated the transcriptome of platelets from prenatally (in utero) THS-exposed mice to delineate their molecular and functional connection with thrombotic events. Our findings identified multiple altered transcripts among coding genes and miRNAs that significantly expanded our current understanding of thrombus development in prenatal THS exposure contexts.

Our analysis revealed 18 miRNAs and 517 coding genes whose expression levels strongly linked prenatal THS exposure to platelet function, and the integrative miRNA-mRNA analysis identified 120 interactions between them. Functional enrichment analysis of all DEGs highlighted activation and signaling regulation of circulating platelets in the exposed mice. Mechanistically, it is intriguing that DEGs showed significant enrichment into several signaling pathways linked with canonical platelet roles in hemostasis and thrombosis. Indeed, functional pathway analysis revealed that several THS exposure-related transcripts were enriched into pathways involved in hemostasis, platelet activation, degranulation, and blood coagulation, which provides credence to our hypothesis. In addition, platelet reactivity associated with prenatal THS exposure was linked to molecular alterations affecting multiple cellular mechanisms that include hemostasis, cytoskeleton rearrangements, oxidative stress, and inflammation. In full agreement with our hypothesis, these significantly enriched functional categories and their downstream signaling pathways comprise—perhaps strikingly—almost all signaling pathways that modulate platelet activity. Interestingly, blood coagulation pathways were also significantly enriched according to our data.

A multitude of mechanisms have been suggested for the increased platelet reactivity in the context of smoke exposure, which are summarized elsewhere [57]. Based on our results, it appears that prenatal THS shares some mechanisms with other routes of exposure to tobacco smoke, despite it being an indirect route of exposure. Oxidative stress and accompanying inflammation have been consistently reported as putative mechanisms mediating cigarette smoking-associated platelet reactivity, which is consistent with the numerous oxidative and inflammatory chemicals [58] that are known to trigger these states [59]. For example, superoxide promotes platelet activity by increasing platelet calcium release [60] and inhibiting NO activity [61]. Similarly enhanced oxidative stress and accumulated hydrogen peroxide were also reported in mice following long-term THS exposure [62]. Thus, our findings suggest oxidative stress as a potential mechanism for the prothrombotic phenotype and emphasize several related pathways, including reaction to oxidative stress, response to reactive oxygen species, and glutathione metabolic processes. As an additional line of evidence, our findings highlight a subset of DEGs enriched in pathways that regulate mitochondrial activity, which are pivotal sites in controlling oxidative stress, such as the regulation of cytochrome C release from mitochondria, etc. Several mitochondrial mechanisms have, indeed, been identified as contributors to platelet activation, including the Mitochondrial Permeability Transportation (MPT) mechanism [63]. Increased intramitochondrial calcium levels have also been shown to mediate phosphatidylserine (PS) exposure [64]. Notably, Bahl et al. (2016) provided further evidence for this notion by showing that exposure to THS triggers stress-induced mitochondrial hyper-fusion and elevated mitochondrial membrane potential in cultured stem cells [36].

Cigarette smoke is well established as a potent proinflammatory agent due to its ability to stimulate inflammatory mediators [65]. Furthermore, thrombosis has been intricately linked to inflammation, and multiple investigations have established a connection between oxidative stress [66], inflammation [67], and cigarette smoking-associated platelet activation [68]. Exposure to THS has also been reported to result in alterations in the plasma proteome that are associated with inflammatory response and elevated urinary oxidative stress markers [26]. Interestingly, some of the impacted transcripts in platelets were significantly enriched in the signaling categories mediating inflammatory response and the immune process, including the chemokine signaling pathway, TNF signaling pathway, regulation of the immune system process, and regulation of leukocyte-mediated immunity. These findings provide more evidence for the importance of tobacco smoke-induced inflammation in the priming of platelets and suggest that this may be one of the mechanisms through which prenatally exposed animals could develop a thrombogenic phenotype.

Although different platelet receptors have different initial signaling mechanisms, they eventually merge into common signaling events that stimulate platelet granule secretion, eventually activating integrin GPIIb-IIIa [69]. For instance, the immunoreceptor tyrosine-based activation motif (ITAM) signaling pathway and phosphoinositide 3-kinases (PI3Ks) are both involved in signal transduction through the glycoprotein Ib-IX-V complex (GPIb- IX), GPVI, and integrins [69]. Furthermore, practically all agonists cause PLC activation. Phosphatidylinositol 4,5-bisphosphate is hydrolyzed by PLC to produce inositol triphosphate (IP3) and diacylglycerol (DAG), which stimulate calcium mobilization and protein kinase C (PKC), respectively, leading to Rap1 GEF CalDAG-GEF1 activation [70]. Additionally, the PI3K/Akt signaling pathway is crucial for platelet activation [71]. Our RNA profiling results showed that six signaling pathways such as control of G-protein activation linked to the signal transduction mediators were significantly enriched in the platelets of prenatally THS-exposed mice, which, in part, further explains the elevated platelet activation.

Our transcriptomic analysis further suggests that prenatal exposure to THS activates the mitogen-activated protein kinase (MAPK) signaling pathway and its downstream effector in circulating platelets, highlighting its potential contribution to the observed thrombogenesis. In fact, platelets contain numerous MAPK pathway components [72], and agonist stimulation activates at least three of the MAPKs, including ERK1/ERK2 [73], some of which are essential for granule secretion [74].

When exposed to diverse biological agonists and stimuli, the platelet cytoskeleton (e.g., actin filaments) is disassembled and reorganized, in part via Rho GTPases [75], resulting in the formation of lamellipodia and filopodia. Our data highlight Rho GTPases and cytoskeleton (actin filament) organization as significantly enriched, which is consistent with our hypothesis.

Since platelets have no nucleus, transcriptional and replicational controls over mRNA expression are ineffective, suggesting a critical role for post-transcriptional regulation of mRNA via platelet miRNAs [56]. Therefore, we performed an integrated analysis of differentially expressed miRNA and mRNA, anticipating that our miRNA–mRNA co-expressed pairs, especially those involving predicted targets, would describe gene regulatory networks that are primarily localized to platelets. Indeed, in support of this notion, our data revealed that post-transcriptional silencing by small RNAs and post-transcriptional regulation of gene expression were significantly enriched pathways in the THS-exposed platelets. Importantly, our study is the first to systematically integrate miRNA-seq and mRNA-seq data from the same set of pure mouse platelets, which allowed for the discovery of 120 miRNA–mRNA interactions underlying platelet thrombogenicity and represents a significant advance in the field. miRNAs miR-144-3p and miR-144-5p were downregulated the most according to our data set. According to reports, increased atherogenesis was seen in the miR-144 knockout (KO) mice, which was linked to its capacity to target vimentin. Moreover, the levels of circulating and tissue vimentin (mostly in the vascular wall) increased after miR-144 ablation but dropped after intravenous miR-144 injection [76]. Interestingly, the exposed platelets exhibited a dramatic reduction in miR-144 levels, which was accompanied by an increase in vimentin levels according to our data set. Additionally, it was discovered that miR-144-3p and 144-5p were downregulated in hospitalized COVID patients [77], who were known to have proactive platelets [78].

Our results additionally suggest that the downregulation of miR-144-3p is concomitant with the upregulation of the CXCL12 chemokine. To this end, recent evidence shows that CXCL12 enhances collagen-induced platelet aggregation [55]. We also found that serum- and glucocorticoid-inducible kinase 1 (SGK1) is a potential target for miR-144-3p (potentially targeted by other miRs according to our data), in addition to being overexpressed in THS-exposed platelets. Evidence shows that SGK1 regulates calcium-dependent platelet function and a host of platelet functional responses (e.g., phosphatidylserine exposure) [79]. Our study also identified miR-486-5p as a downregulated miRNA that targets specific genes related to platelet function and activation. It is also implicated in the pathogenesis of immune thrombocytopenia (ITP) and may function as a diagnostic and predictive biomarker for the disorder. Its expression in platelets changes during storage, indicating its significance in platelet quality and viability. Our data set indicates that miR-486a-5p potentially targets phospholipase D1 (PLD1), which regulates platelet activity by modulating αIIbβ3 integrin activation and α-granule release [80].

Notably, we found that the majority of the miRNAs whose expression was altered were downregulated. This global reduction in the expression of miRNAs has been previously reported in alveolar macrophages of smokers [81]. In addition, downregulation was shown to be the primary alteration in miRNA expression in the lungs of mice exposed to cigarette smoke [82]. We also found the downregulated mRNAs to be more prevalent than the upregulated mRNAs in the exposed platelets. Indeed, a systemic downregulation of both miRNA and mRNA in circulating platelets occurs with aging, which is characterized by the robust production of extracellular vesicles that are laden with mRNAs and miRNAs and may lead to heightened reactivity and aggregation [83]. This phenotype is accompanied by a generalized reduction in the levels of these molecules in the circulating platelets and is coupled with the buildup of reactive oxygen species [84]. Given that cigarette smoke, as well as THS exposure, acutely promotes oxidative stress and chronic smoking leads to systemic inflammation [85], this could be associated with similarly enhanced secretion of extracellular vesicles that are loaded with mRNA and miRNA, which could explain the greater number of downregulation events observed post THS exposure. Another study found that exposure to particulate matter (PM) increased the release of extracellular vesicles, including those mainly produced by platelets [86]. It is worth noting that specific miRNA species linked to CVD fibrinogen levels were found to be downregulated as a result of this increased release of extracellular vesicles [86].

We also sought to predict the potential chemical components of the toxic profile of THS that could be triggered by chemical–gene interactions using a toxicogenomic approach. Our data demonstrated that the majority of the differentially expressed genes are, in fact, linked to one or more of the top 10 chemicals generated by THS, although some of these chemical–gene interactions have been previously reported in different contexts. For example, benzo[a]pyrene exposure has been reported to change the expression of oxidative stress genes such as UCP2 and TNF-α1, increasing the intracellular reactive oxygen species in mouse embryo fibroblast cells [87] and perturbed CXCL12 expression in human T-lymphocytes [88].

One of the strengths of our study is that we employed—for the first time—integrated miRNA–mRNA pairing coupled with bioinformatic analysis to highlight the molecular basis of prenatal THS-associated platelet hyperactivity and thrombogenicity. Another strength involves using the same population of purified platelets depleted from other blood cells to generate both mRNA and small RNA libraries for sequencing, which guaranteed the temporal and spatial co-localization of mRNA and miRNA that is required for the identification of the corresponding gene regulatory networks. We were also able to provide an external replication of our transcriptomic data to support the validity of our findings. In fact, we verified the expression of the majority of altered transcripts (mRNAs and miRNAs) on a distinct cohort of individual mice using a different approach (Q-PCR).

We also recognize that the current study has some shortcomings. Since a single mouse cannot provide enough RNA for RNA sequencing, we had to pool platelets from several mice; nonetheless, this was mitigated by the Q-PCR and the use of a different mouse cohort. We also acknowledge that the mice used in this work were all male; hence, we do not know if sex effects exist, which is the scope of a future investigation.

In conclusion, our findings document—for the first time—that prenatal/in utero THS exposure drastically and globally alters the platelet transcriptome of offspring mice, which appears to contribute to their thrombogenic phenotype. In addition, we identified a host of mRNA–miRNA pairs that could potentially be involved.

## 4. Materials and Methods

### 4.1. Animals and Exposure

C57BL/6J mice were purchased from Jackson Laboratory (Bar Harbor, ME, USA). Upon arrival, males were single-housed, whereas females were housed in groups of two per cage for one week for acclimation prior to mating and before subjecting the latter to our previously described and validated exposure protocol [22,25,27] using the Teague Enterprises Smoking Machine. Since the detrimental health effects of maternal smoke have been shown to endure for months, years, or even into adulthood in some cases [89], the sampling and experimentation of mice started at 10–12 weeks old.

### 4.2. Platelet Purification and RNA Extraction

Whole blood was collected by cardiac puncture into 10% anticoagulant consisting of 3.8% sodium citrate and 0.4 mM of Gly-Pro-Arg-Pro (GPRP). Platelets were purified using an iohexol gradient medium as previously described [90]. To ensure the depletion of white blood cells and red blood cells, we used CD45- and Ter-119-coated magnetic particles (Miltenyi Biotec, Gaithersburg, MD, USA), as directed by the manufacturer. Platelets were then lysed in a suitable volume of TRIzol reagent (Invitrogen, Carlsbad, CA, USA), and the RNA extraction process was performed as per the manufacturer’s procedure.

### 4.3. mRNA Sequencing and Analysis

Isolated total RNA from purified platelets for both maternal THS-exposed and control samples was assessed for integrity via Agilent RNA Tape on a TapeStation 4200 quantified via Qubit fluorometric assay and normalized for library preparation. The mRNA sequencing libraries were generated via the Illumina TruSeq stranded kit (Illumina, San Diego, CA, USA), uniquely indexed, and sequenced at 100 million raw reads per sample on a paired end 2 × 75 sequencing run on an Illumina NextSeq 550 (Illumina, San Diego, CA, USA). The raw reads were trimmed using Trimmomatic (v.0.22) and aligned to the reference genome using Kallisto software (version mm10). Differential expression and significance were determined by utilizing Voom normalization and the Limma R package (version 3.50.0) with a false discovery rate (FDR) according to Benjamini-Hochberg [91].

### 4.4. miRNA Sequencing and Analysis

Small RNA libraries were generated using the Bioo Scientific NextFlex Small RNA v3 kit, indexed, then sequenced at 100 million raw reads per sample on an Illumina NextSeq 550. Preprocessed reads were mapped to a mouse genome assembly (version mm10) with the qAlign function (QuasR package “version 1.34.0”) aligner parameter set to a default (Bowtie). To capture miRNA encoded by multiple genes and avoid their underestimation, we set the maxHits parameter in the qAlign function to 50 hits in the genome. The coordinate of mature miRNAs was imported from miRbase, subjected to extension by 3 bp on each side, and passed to the qCount function of the QuasR package (version 1.34.0), which quantifies the number of reads within any mature miRNA. Data was filtered, then normalized using the edgeR package (version 3.36.0), and to identify the differential expressed miRNAs, the R Limma package (version 3.50.0) was used.

### 4.5. Functional Enrichment Analysis

For the annotation and pathway enrichment of the candidate genes from differentially expressed gene (DEG) analysis, we used the following accessible public databases: Gene Ontology (GO) [92]; the Kyoto Encyclopedia of Genes and Genomes (KEGG) [93]; and the database of reactions, pathways, and biological processes (Reactome) [94] through DAVID (https://david.ncifcrf.gov, accessed on 30 April 2024). Terms with FDR < 0.05 were regarded as statistically significant.

### 4.6. Identification of miRNA-Targeted Genes and the mRNA–miRNA Regulatory Network

The MultiMiR R package [95] was used to identify interactions between DE-mRNA and DE-miRNAs using get_multimir() function. The miRNA-mRNA network was built according to the following criteria: (1) differential expression of target genes (FDR < 0.05 and FC > 1.5 or <−1.5); (2) opposite direction of FC of mRNA and miRNA between in utero THS-exposed and control mice. Databases integrated in the process include miRecords, miRTarBase, TarBase, DIANA-microT, ElMMo, MicroCosm, miRanda, miRDB, PicTar, PITA, TargetScan, miR2Disease, PharmacomiR, and PhenomiR.

### 4.7. Validation of DEGs and DE miRs by Quantitative RT-qPCR

RNA was isolated from 40 individual mice of a distinct exposure cohort (20 in utero THS- and 20 CA-exposed) using TRIzol reagent (Invitrogen, USA) for the purpose of validating our RNA seq findings. mRNA and miRNA were reverse-transcribed with a QuanitNova Reverse Transcription Kit and miRCURY LNA RT Kit (Qiagen, Germantown, MD, USA), respectively, following the manufacturers’ recommendations. Quantitative real-time PCR (qPCR) measurement was performed for mRNA and miRNA using a QuantiNova SYBR Green PCR Kit and miRCURY SYBR Green PCR Kit (Qiagen, USA). Primers were synthesized by Integrated DNA Technologies (Coralville, IA, USA), and the primer sequences are available in Supplementary Table S1. The levels of transcripts for mRNA were normalized to housekeeping genes β-Actin (ACTB), β2-microglobulin (B2M), and Ribosomal Protein Lateral talk subunit P0 (RPLP0), whereas miRNAs were normalized to RNU1A1 and 5S rRNA. The relative mRNA expression was plotted as fold changes calculated by the 2^−∆∆Ct^ method [96].

### 4.8. Toxic Chemical–Gene Interaction Enrichment Analysis Using the Toxicogenomics Database

The Comparative Toxicogenomics Database (CTD) is a comprehensive platform that integrates extensive data on chemical substances, genes, functional phenotypes, and disease interactions [97]. The CTD significantly facilitates research into environmental factors related to diseases and their potential mechanisms. We input a list of differentially expressed genes (DEGs) into the CTD to identify toxic substances closely associated with these genes. The results were generated in a CSV file, which was then filtered based on the THS toxic profile, then used to create illustrative figures depicting the relationships between genes and chemicals.

### 4.9. Statistical Analysis

We used R statistical programming language version 4.1.0 with the following packages: Limma (version 3.52.4), edgeR (version 3.36.0), ggplot2 (version 3.4.0), circlize (version 0.4.15), MultiMiR, and QuasR (version 1.34.0). The qPCR data analysis was performed using GraphPad PRISM V7 statistical software and presented as mean ± SD log2 fold change, and the statistical analysis was performed using Student’s *t*-test at a 5% significance level.

## Figures and Tables

**Table 1:** The most significantly enriched pathways according to the Reactome database.

Category	Term	FDR
REACTOME_PATHWAY	R-MMU-168256~Immune System	1.9 × 10^−5^
REACTOME_PATHWAY	R-MMU-109582~Hemostasis	1.9 × 10^−5^
REACTOME_PATHWAY	R-MMU-2262752~Cellular responses to stress	1.9 × 10^−5^
REACTOME_PATHWAY	R-MMU-8953897~Cellular responses to stimuli	1.9 × 10^−5^
REACTOME_PATHWAY	R-MMU-76002~Platelet activation, signaling and aggregation	1.0 × 10^−4^
REACTOME_PATHWAY	R-MMU-9716542~Signaling by Rho GTPases, Miro GTPases and RHOBTB3	6.3 × 10^−4^
REACTOME_PATHWAY	R-MMU-194315~Signaling by Rho GTPases	6.4 × 10^−4^
REACTOME_PATHWAY	R-MMU-9711123~Cellular response to chemical stress	1.1 × 10^−3^
REACTOME_PATHWAY	R-MMU-9006925~Intracellular signaling by second messengers	1.9 × 10^−3^
REACTOME_PATHWAY	R-MMU-1257604~PIP3 activates AKT signaling	5.8 × 10^−3^
REACTOME_PATHWAY	R-MMU-5683057~MAPK family signaling cascades	1.5 × 10^−2^
REACTOME_PATHWAY	R-MMU-114604~GPVI-mediated activation cascade	3.2 × 10^−2^
REACTOME_PATHWAY	R-MMU-9012999~RHO GTPase cycle	3.2 × 10^−2^
REACTOME_PATHWAY	R-MMU-5673001~RAF/MAP kinase cascade	3.6 × 10^−2^
REACTOME_PATHWAY	R-MMU-76005~Response to elevated platelet cytosolic Ca^2+^	4.3 × 10^−2^
REACTOME_PATHWAY	R-MMU-5684996~MAPK1/MAPK3 signaling	4.5 × 10^−2^
REACTOME_PATHWAY	R-MMU-2029482~Regulation of actin dynamics for phagocytic cup formation	5.1 × 10^−2^
REACTOME_PATHWAY	R-MMU-199418~Negative regulation of the PI3K/AKT network	5.9 × 10^−2^
REACTOME_PATHWAY	R-MMU-5675221~Negative regulation of MAPK pathway	7.4 × 10^−2^
REACTOME_PATHWAY	R-MMU-983231~Factors involved in megakaryocyte development and platelet production	8.4 × 10^−2^
REACTOME_PATHWAY	R-MMU-195258~RHO GTPase Effectors	8.7 × 10^−2^
REACTOME_PATHWAY	R-MMU-114608~Platelet degranulation	1.1 × 10^−1^

**Table 2:** Q-PCR validation for the selected transcripts (mRNA) from RNA seq data set.

#	Gene ID	RNA SEQ			Q-PCR			
		Log2FC	Trend	*p* Value	Log2FC	Trend	*p* Value	SIG
1	PLD1	2.25	UP	0.0107	2.14	UP	0.00022	S
2	MEOX2	1.89	UP	0.0316	1.00	UP	0.07618	NS ↑
3	EPAS1	1.09	UP	0.0021	0.72	UP	0.07022	NS ↑
4	ABLIM1	1.18	UP	0.0187	0.75	UP	0.05965	NS ↑
5	CNN3	1.03	UP	0.0267	1.45	UP	0.01718	S
6	VIM	0.98	UP	0.0065	1.08	UP	0.00311	S
7	ARHGEF15	0.92	UP	0.0137	1.65	UP	0.01669	S
8	CXCL12	0.62	UP	0.0037	1.26	UP	0.00109	S
9	GDE1	0.50	UP	0.0448	0.53	UP	0.01594	S
10	PTEN	−0.74	Down	0.0114	−0.89	Down	0.00118	S
11	KLF6	−0.80	Down	0.0046	−0.88	Down	0.00321	S
12	TRAF5	−1.32	Down	0.0130	−1.57	Down	0.00305	S
13	GPX1	−1.62	Down	0.0037	−1.67	Down	0.00130	S
14	TFRC	−1.82	Down	0.0326	−1.87	Down	0.00104	S
15	UCP2	−2.59	Down	0.0042	−2.05	Down	0.00030	S
16	CD82	−2.76	Down	0.0139	−0.25	Down	0.26002	NS
17	TMOD1	−3.32	Down	0.0174	−2.33	Down	0.00916	S
18	TSPAN33	−3.39	Down	0.0097	−3.27	Down	0.00024	S

The upward arrow ↑ indicated that the gene displayed an upregulated trend, although it did not reach statistical significance.

**Table 3:** Q-PCR validation for the selected transcripts (miRNA) from RNA seq data set.

#	Gene ID	RNA SEQ			Q-PCR			
		Log2FC	Trend	*p* Value	Log2FC	Trend	*p* Value	SIG
1	mmu-miR-320-3p	0.51	UP	0.0132	0.96	UP	0.0205	S
2	mmu-miR-674-5p	0.61	UP	0.0313	1.26	UP	0.00326	S
3	mmu-miR-7a-5p	−0.73	Down	0.0227	−1.46	Down	0.0027	S
4	mmu-miR-30a-5p	−1.03	Down	0.0061	−1.15	Down	0.0713	NS ↓
5	mmu-miR-30e-5p	−0.70	Down	0.0061	−1.10	Down	0.1053	NS ↓
6	mmu-miR-33-5p	−0.94	Down	0.0130	−1.70	Down	0.0146	S
7	mmu-miR-144-3p	−4.69	Down	0.0002	−3.96	Down	1.6 × 10^−6^	S
8	mmu-miR-144-5p	−3.53	Down	0.0031	−3.24	Down	2.7 × 10^−5^	S
9	mmu-miR-486a-3p	−2.79	Down	0.0061	−1.53	Down	0.0015	S

The downward arrow ↓ signifies that the gene exhibited a downregulated trend, although it did not reach statistical significance.

## Data Availability

Requests for data will be honored on a case-by-case basis, and data will be provided by the corresponding author.

## Supplementary Materials

The supporting information can be downloaded at: https://www.mdpi.com/article/10.3390/ijms26157633/s1.

## Abbreviations

The following abbreviations are used in this manuscript:

THS	Thirdhand smoke
CA	Clean air
GPRP	Gly-Pro-Arg-Pro
DEG	Differentially Expressed Gene
KEGG	Kyoto Encyclopedia of Genes and Genomes
CTD	Comparative Toxicogenomics Database
PCA	Principal Component Analysis

## References

[1] Davis J.W., Shelton L., Watanabe I.S., Arnold J. (1989). Passive smoking affects endothelium and platelets. Arch. Intern. Med..

[2] Protano C., Vitali M. (2011). The New Danger of Thirdhand Smoke: Why Passive Smoking Does Not Stop at Secondhand Smoke. Environ. Health Perspect..

[3] Sleiman M., Gundel L.A., Pankow J.F., Jacob P., Singer B.C., Destaillats H. (2010). Formation of carcinogens indoors by surface-mediated reactions of nicotine with nitrous acid, leading to potential *thirdhand smoke* hazards. Proc. Natl. Acad. Sci. USA.

[4] Sleiman M., Logue J.M., Luo W., Pankow J.F., Gundel L.A., Destaillats H. (2014). Inhalable Constituents of Thirdhand Tobacco Smoke: Chemical Characterization and Health Impact Considerations. Environ. Sci. Technol..

[5] Becquemin M.H., Bertholon J.F., Bentayeb M., Attoui M., Ledur D., Roy F., Roy M., Annesi-Maesano I., Dautzenberg B. (2010). Third-hand smoking: Indoor measurements of concentration and sizes of cigarette smoke particles after resuspension. Tob. Control.

[6] Matt G.E., Quintana P.J.E., Hovell M.F., Chatfield D., Ma D.S., Romero R., Uribe A. (2008). Residual tobacco smoke pollution in used cars for sale: Air, dust, and surfaces. Nicotine Tob. Res..

[7] Matt G.E., Quintana P.J.E., Zakarian J.M., Fortmann A.L., Chatfield D.A., Hoh E., Uribe A.M., Hovell M.F. (2010). When smokers move out and non-smokers move in: Residential thirdhand smoke pollution and exposure. Tob. Control.

[8] Schick S. (2010). Thirdhand smoke: Here to stay. Tob. Control.

[9] Winickoff J.P., Friebely J., Tanski S.E., Sherrod C., Matt G.E., Hovell M.F., McMillen R.C. (2009). Beliefs About the Health Effects of “Thirdhand” Smoke and Home Smoking Bans. Pediatrics.

[10] Destaillats H., Singer B.C., Gundel L.A. (2007). Evidence of acid-base interactions between amines and model indoor surfaces by ATR-FTIR spectroscopy. Atmos. Environ..

[11] Sleiman M., Destaillats H., Smith J.D., Liu C.L., Ahmed M., Wilson K.R., Gundel L.A. (2010). Secondary organic aerosol formation from ozone-initiated reactions with nicotine and secondhand tobacco smoke. Atmos. Environ..

[12] Kuschner W.G., Reddy S., Mehrotra N., Paintal H.S. (2011). Electronic cigarettes and thirdhand tobacco smoke: Two emerging health care challenges for the primary care provider. Int. J. Gen. Med..

[13] Matt G.E., Quintana P.J.E., Hovell M.F., Bernert J.T., Song S., Novianti N., Juarez T., Floro J., Gehrman C., Garcia M. (2004). Households contaminated by environmental tobacco smoke: Sources of infant exposures. Tob. Control.

[14] Adhami N., Starck S.R., Flores C., Martins Green M. (2016). A Health Threat to Bystanders Living in the Homes of Smokers: How Smoke Toxins Deposited on Surfaces Can Cause Insulin Resistance. PLoS ONE.

[15] Prignot J.J. (2011). Recent Contributions of Air- and Biomarkers to the Control of Secondhand Smoke (SHS): A Review. Int. J. Environ. Res. Public Health.

[16] Martins-Green M., Adhami N., Frankos M., Valdez M., Goodwin B., Lyubovitsky J., Dhall S., Garcia M., Egiebor I., Martinez B. (2014). Cigarette Smoke Toxins Deposited on Surfaces: Implications for Human Health. PLoS ONE.

[17] Burton A. (2011). Does the Smoke Ever Really Clear? Thirdhand Smoke Exposure Raises New Concerns. Environ. Health Perspect..

[18] Chaouachi K. (2009). Hookah (Shisha, Narghile) Smoking and Environmental Tobacco Smoke (ETS). A critical review of the relevant literature and the public health consequences. Int. J. Environ. Res. Public Health.

[19] Drehmer J.E., Ossip D.J., Rigotti N.A., Nabi-Burza E., Woo H., Wasserman R.C., Chang Y., Winickoff J.P. (2012). Pediatrician Interventions and Thirdhand Smoke Beliefs of Parents. Am. J. Prev. Med..

[20] Whitlatch A., Schick S. (2018). Thirdhand Smoke at Philip Morris. Nicotine Tob. Res..

[21] Thomas J.L., Hecht S.S., Luo X., Ming X., Ahluwalia J.S., Carmella S.G. (2013). Thirdhand Tobacco Smoke: A Tobacco-Specific Lung Carcinogen on Surfaces in Smokers’ Homes. Nicotine Tob. Res..

[22] Karim Z.A., Alshbool F.Z., Vemana H.P., Adhami N., Dhall S., Espinosa E.V., Martins-Green M., Khasawneh F.T. (2015). Third-hand Smoke: Impact on Hemostasis and Thrombogenesis. J. Cardiovasc. Pharmacol..

[23] Godwin M.D., Aggarwal A., Hilt Z., Shah S., Gorski J., Cameron S.J. (2022). Sex-Dependent Effect of Platelet Nitric Oxide: Production and Platelet Reactivity in Healthy Individuals. JACC Basic Transl. Sci..

[24] Snijders A.M., Zhou M., Whitehead T.P., Fitch B., Pandey P., Hechmer A., Huang A., Schick S.F., de Smith A.J., Olshen A.B. (2021). In utero and early-life exposure to thirdhand smoke causes profound changes to the immune system. Clin. Sci..

[25] Ali H.E., Alarabi A.B., Karim Z.A., Rodriguez V., Hernandez K.R., Lozano P.A., El-Halawany M.S., Alshbool F.Z., Khasawneh F.T. (2021). In utero thirdhand smoke exposure modulates platelet function in a sex-dependent manner. Haematologica.

[26] Sakamaki-Ching S., Schick S., Grigorean G., Li J., Talbot P. (2022). Dermal thirdhand smoke exposure induces oxidative damage, initiates skin inflammatory markers, and adversely alters the human plasma proteome. EBioMedicine.

[27] Villalobos-García D., Ali H.E.A., Alarabi A.B., El-Halawany M.S., Alshbool F.Z., Khasawneh F.T. (2022). Exposure of Mice to Thirdhand Smoke Modulates In Vitro and In Vivo Platelet Responses. Int. J. Mol. Sci..

[28] Bahl V., Shim H.J., Jacob P., Dias K., Schick S.F., Talbot P. (2016). Thirdhand smoke: Chemical dynamics, cytotoxicity, and genotoxicity in outdoor and indoor environments. Toxicol. In Vitro.

[29] Xu B., Chen M., Yao M., Ji X., Mao Z., Tang W., Qiao S., Schick S.F., Mao J.-H., Hang B. (2015). Metabolomics reveals metabolic changes in male reproductive cells exposed to thirdhand smoke. Sci. Rep..

[30] Brody J.S. (2012). Transcriptome alterations induced by cigarette smoke. Int. J. Cancer.

[31] Boyle J.O., Gümüş Z.H., Kacker A., Choksi V.L., Bocker J.M., Zhou X.K., Yantiss R.K., Hughes D.B., Du B., Judson B.L. (2010). Effects of cigarette smoke on the human oral mucosal transcriptome. Cancer Prev. Res..

[32] Harvey B.-G., Heguy A., Leopold P.L., Carolan B.J., Ferris B., Crystal R.G. (2007). Modification of gene expression of the small airway epithelium in response to cigarette smoking. J. Mol. Med..

[33] Huan T., Joehanes R., Schurmann C., Schramm K., Pilling L.C., Peters M.J., Mägi R., DeMeo D., O’Connor G.T., Ferrucci L. (2016). A whole-blood transcriptome meta-analysis identifies gene expression signatures of cigarette smoking. Hum. Mol. Genet..

[34] Pickett G., Seagrave J., Boggs S., Polzin G., Richter P., Tesfaigzi Y. (2010). Effects of 10 cigarette smoke condensates on primary human airway epithelial cells by comparative gene and cytokine expression studies. Toxicol. Sci..

[35] López-Boado Y.S., Li J.U., Clayton C.L., Wright J.L., Churg A. (2010). Modification of the rat airway explant transcriptome by cigarette smoke. Inhal. Toxicol..

[36] Bahl V., Johnson K., Phandthong R., Zahedi A., Schick S.F., Talbot P. (2016). From the Cover: Thirdhand Cigarette Smoke Causes Stress-Induced Mitochondrial Hyperfusion and Alters the Transcriptional Profile of Stem Cells. Toxicol. Sci..

[37] Quelhas D., Kompala C., Wittenbrink B., Han Z., Parker M., Shapiro M., Downs S., Kraemer K., Fanzo J., Morris S. (2018). The association between active tobacco use during pregnancy and growth outcomes of children under five years of age: A systematic review and meta-analysis. BMC Public Health.

[38] McEvoy C.T., Spindel E.R. (2017). Pulmonary Effects of Maternal Smoking on the Fetus and Child: Effects on Lung Development, Respiratory Morbidities, and Life Long Lung Health. Paediatr. Respir. Rev..

[39] Noakes P.S., Hale J., Thomas R., Lane C., Devadason S.G., Prescott S.L. (2006). Maternal smoking is associated with impaired neonatal toll-like-receptor-mediated immune responses. Eur. Respir. J..

[40] Polańska K., Jurewicz J., Hanke W. (2015). Smoking and alcohol drinking during pregnancy as the risk factors for poor child neurodevelopment—A review of epidemiological studies. Int. J. Occup. Med. Environ. Health.

[41] Wiklund P., Karhunen V., Richmond R.C., Parmar P., Rodriguez A., De Silva M., Wielscher M., Rezwan F.I., Richardson T.G., Veijola J. (2019). DNA methylation links prenatal smoking exposure to later life health outcomes in offspring. Clin. Epigenetics.

[42] Joubert B.R., Felix J.F., Yousefi P., Bakulski K.M., Just A.C., Breton C., Reese S.E., Markunas C.A., Richmond R.C., Xu C.-J. (2016). DNA Methylation in Newborns and Maternal Smoking in Pregnancy: Genome-wide Consortium Meta-analysis. Am. J. Hum. Genet..

[43] Everson T.M., Vives-Usano M., Seyve E., Cardenas A., Lacasaña M., Craig J.M., Lesseur C., Baker E.R., Fernandez-Jimenez N., Heude B. (2021). Placental DNA methylation signatures of maternal smoking during pregnancy and potential impacts on fetal growth. Nat. Commun..

[44] Chhabra D., Sharma S., Kho A.T., Gaedigk R., Vyhlidal C.A., Leeder J.S., Morrow J., Carey V.J., Weiss S.T., Tantisira K.G. (2014). Fetal lung and placental methylation is associated with in utero nicotine exposure. Epigenetics.

[45] Breton C.V., Byun H.-M., Wenten M., Pan F., Yang A., Gilliland F.D. (2009). Prenatal tobacco smoke exposure affects global and gene-specific DNA methylation. Am. J. Respir. Crit. Care Med..

[46] Rauschert S., Melton P.E., Burdge G., Craig J.M., Godfrey K.M., Holbrook J.D., Lillycrop K., Mori T.A., Beilin L.J., Oddy W.H. (2019). Maternal Smoking During Pregnancy Induces Persistent Epigenetic Changes into Adolescence, Independent of Postnatal Smoke Exposure and Is Associated with Cardiometabolic Risk. Front. Genet..

[47] Bray P.F., McKenzie S.E., Edelstein L.C., Nagalla S., Delgrosso K., Ertel A., Kupper J., Jing Y., Londin E., Loher P. (2013). The complex transcriptional landscape of the anucleate human platelet. BMC Genom..

[48] Mills E.W., Green R., Ingolia N.T. (2017). Slowed decay of mRNAs enhances platelet specific translation. Blood.

[49] Brown G.T., Narayanan P., Li W., Silverstein R.L., McIntyre T.M. (2013). Lipopolysaccharide stimulates platelets through an IL-1β autocrine loop. J. Immunol..

[50] Shiva S., Novelli E.M., Bullock G.C., Kenny E., Hill G., Corey C.G. (2014). Mitochondrial Uncoupling Protein 2 Is Present in Human Platelets and Regulates Platelet Activation. Blood.

[51] Englert M., Aurbach K., Becker I.C., Gerber A., Heib T., Wackerbarth L.M., Kusch C., Mott K., Araujo G.H.M., Baig A.A. (2022). Impaired microtubule dynamics contribute to microthrombocytopenia in RhoB-deficient mice. Blood Adv..

[52] Mangin P., Ohlmann P., Eckly A., Cazenave J., Lanza F., Gachet C. (2004). The P2Y1 receptor plays an essential role in the platelet shape change induced by collagen when TxA2 formation is prevented. J. Thromb. Haemost..

[53] Weng Z., Li D., Zhang L., Chen J., Ruan C., Chen G., Gartner T.K., Liu J. (2010). PTEN regulates collagen-induced platelet activation. Blood.

[54] Poisson C., Rollin S., Véronneau S., Bousquet S.M., Larrivée J.-F., Le Gouill C., Boulay G., Stankova J., Rola-Pleszczynski M. (2009). Caveolae Facilitate but Are Not Essential for Platelet-Activating Factor-Mediated Calcium Mobilization and Extracellular Signal-Regulated Kinase Activation. J. Immunol..

[55] Leberzammer J., Agten S.M., Blanchet X., Duan R., Ippel H., Megens R.T.A., Schulz C., Aslani M., Duchene J., Döring Y. (2022). Targeting platelet derived CXCL12 impedes arterial thrombosis. Blood.

[56] Nagalla S., Shaw C., Kong X., Kondkar A.A., Edelstein L.C., Ma L., Chen J., McKnight G.S., López J.A., Yang L. (2011). Platelet microRNA-mRNA coexpression profiles correlate with platelet reactivity. Blood.

[57] Csordas A., Bernhard D. (2013). The biology behind the atherothrombotic effects of cigarette smoke. Nat. Rev. Cardiol..

[58] Malyla V., Paudel K.R., Shukla S.D., Donovan C., Wadhwa R., Pickles S., Chimankar V., Sahu P., Bielefeldt-Ohmann H., Bebawy M. (2020). Recent advances in experimental animal models of lung cancer. Future Med. Chem..

[59] Salahuddin S., Prabhakaran D., Roy A. (2012). Pathophysiological Mechanisms of Tobacco-Related CVD. Glob. Heart.

[60] Schaeffer G., Wascher T.C., Kostner G.M., Graier W.F. (1999). Alterations in platelet Ca^2+^ signalling in diabetic patients is due to increased formation of superoxide anions and reduced nitric oxide production. Diabetologia.

[61] Spinetti G., Kraenkel N., Emanueli C., Madeddu P. (2008). Diabetes and vessel wall remodelling: From mechanistic insights to regenerative therapies. Cardiovasc. Res..

[62] Adhami N., Chen Y., Martins-Green M. (2017). Biomarkers of disease can be detected in mice as early as 4 weeks after initiation of exposure to third-hand smoke levels equivalent to those found in homes of smokers. Clin. Sci..

[63] Remenyi G., Szasz R., Friese P., Dale G.L. (2005). Role of mitochondrial permeability transition pore in coated-platelet formation. Arterioscler. Thromb. Vasc. Biol..

[64] Choo H.-J., Saafir T.B., Mkumba L., Wagner M.B., Jobe S.M. (2012). Mitochondrial calcium and reactive oxygen species regulate agonist-initiated platelet phosphatidylserine exposure. Arterioscler. Thromb. Vasc. Biol..

[65] Paudel K.R., Panth N., Manandhar B., Singh S.K., Gupta G., Wich P.R., Nammi S., MacLoughlin R., Adams J., Warkiani M.E. (2022). Attenuation of Cigarette-Smoke-Induced Oxidative Stress, Senescence, and Inflammation by Berberine-Loaded Liquid Crystalline Nanoparticles: In Vitro Study in 16HBE and RAW264.7 Cells. Antioxidants.

[66] James R.W., Leviev I., Righetti A. (2000). Smoking is associated with reduced serum paraoxonase activity and concentration in patients with coronary artery disease. Circulation.

[67] Yasue H., Hirai N., Mizuno Y., Harada E., Itoh T., Yoshimura M., Kugiyama K., Ogawa H. (2006). Low-grade inflammation, thrombogenicity, and atherogenic lipid profile in cigarette smokers. Circ. J..

[68] FitzGerald G.A., Oates J.A., Nowak J. (1988). Cigarette smoking and hemostatic function. Am. Heart J..

[69] Li Z., Delaney M.K., O’BRien K.A., Du X. (2010). Signaling during platelet adhesion and activation. Arterioscler. Thromb. Vasc. Biol..

[70] Crittenden J.R., Bergmeier W., Zhang Y., Piffath C.L., Liang Y., Wagner D.D., Housman D.E., Graybiel A.M. (2004). CalDAG-GEFI integrates signaling for platelet aggregation and thrombus formation. Nat. Med..

[71] Chen Z., Li T., Kareem K., Tran D., Griffith B.P., Wu Z.J. (2019). The role of PI3K/Akt signaling pathway in non-physiological shear stress-induced platelet activation. Artif. Organs.

[72] Börsch-Haubold A.G., Kramer R.M., Watson S.P. (1996). Inhibition of mitogen-activated protein kinase kinase does not impair primary activation of human platelets. Biochem. J..

[73] Papkoff J., Chen R.-H., Blenis J., Forsman J. (1994). p42 mitogen-activated protein kinase and p90 ribosomal S6 kinase are selectively phosphorylated and activated during thrombin-induced platelet activation and aggregation. Mol. Cell Biol..

[74] Adam F., Kauskot A., Nurden P., Sulpice E., Hoylaerts M.F., Davis R.J., Rosa J.-P., Bryckaert M. (2010). Platelet JNK1 is involved in secretion and thrombus formation. Blood.

[75] Aslan J.E., Mccarty O.J.T. (2013). Rho GTPases in platelet function. J. Thromb. Haemost..

[76] He Q., Wang F., Honda T., Greis K.D., Redington A.N. (2020). Ablation of miR-144 increases vimentin expression and atherosclerotic plaque formation. Sci. Rep..

[77] Madè A., Greco S., Vausort M., Miliotis M., Schordan E., Baksi S., Zhang L., Baryshnikova E., Ranucci M., Cardani R. (2022). Association of miR-144 levels in the peripheral blood with COVID-19 severity and mortality. Sci. Rep..

[78] Zhang S., Liu Y., Wang X., Yang L., Li H., Wang Y., Liu M., Zhao X., Xie Y., Yang Y. (2020). SARS-CoV-2 binds platelet ACE2 to enhance thrombosis in COVID-19. J. Hematol. Oncol..

[79] Yang H.-Y., Zhang C., Hu L., Liu C., Pan N., Li M., Han H., Zhou Y., Li J., Zhao L.-Y. (2022). Platelet CFTR inhibition enhances arterial thrombosis via increasing intracellular Cl^−^ concentration and activation of SGK1 signaling pathway. Acta Pharmacol. Sin..

[80] Elvers M., Stegner D., Hagedorn I., Kleinschnitz C., Braun A., Kuijpers M.E.J., Boesl M., Chen Q., Heemskerk J.W.M., Stoll G. (2010). Impaired alpha(IIb)beta(3) integrin activation and shear-dependent thrombus formation in mice lacking phospholipase D1. Sci Signal..

[81] Graff J.W., Powers L.S., Dickson A.M., Kim J., Reisetter A.C., Hassan I.H., Kremens K., Gross T.J., Wilson M.E., Monick M.M. (2012). Cigarette smoking decreases global microRNA expression in human alveolar macrophages. PLoS ONE.

[82] Izzotti A., Calin G.A., Steele V.E., Croce C.M., De Flora S. (2009). Relationships of microRNA expression in mouse lung with age and exposure to cigarette smoke and light. FASEB J..

[83] Espinosa-Parrilla Y., Gonzalez-Billault C., Fuentes E., Palomo I., Alarcón M. (2019). Decoding the Role of Platelets and Related MicroRNAs in Aging and Neurodegenerative Disorders. Front. Aging Neurosci..

[84] Donato A.J., Walker A.E., Magerko K.A., Bramwell R.C., Black A.D., Henson G.D., Lawson B.R., Lesniewski L.A., Seals D.R. (2013). Life-long caloric restriction reduces oxidative stress and preserves nitric oxide bioavailability and function in arteries of old mice. Aging Cell.

[85] Yanbaeva D.G., Dentener M.A., Creutzberg E.C., Wesseling G., Wouters E.F.M. (2007). Systemic effects of smoking. Chest.

[86] Pergoli L., Cantone L., Favero C., Angelici L., Iodice S., Pinatel E., Hoxha M., Dioni L., Letizia M., Albetti B. (2017). Extracellular vesicle-packaged miRNA release after short-term exposure to particulate matter is associated with increased coagulation. Part. Fibre Toxicol..

[87] Omidian K., Rafiei H., Bandy B. (2017). Polyphenol inhibition of benzo[a]pyrene-induced oxidative stress and neoplastic transformation in an in vitro model of carcinogenesis. Food Chem. Toxicol..

[88] Liamin M., Le Mentec H., Evrard B., Huc L., Chalmel F., Boutet-Robinet E., Le Ferrec E., Sparfel L. (2018). Genome-Wide Transcriptional and Functional Analysis of Human T Lymphocytes Treated with Benzo[α]pyrene. Int. J. Mol. Sci..

[89] Gall S., Huynh Q.L., Magnussen C.G., Juonala M., Viikari J.S., Kähönen M., Dwyer T., Raitakari O.T., Venn A. (2014). Exposure to parental smoking in childhood or adolescence is associated with increased carotid intima-media thickness in young adults: Evidence from the Cardiovascular Risk in Young Finns study and the Childhood Determinants of Adult Health Study. Eur. Hear. J..

[90] Narciso M.G., Nasimuzzaman M. (2019). Purification of Platelets from Mouse Blood. J. Vis. Exp..

[91] Ritchie M.E., Phipson B., Wu D., Hu Y., Law C.W., Shi W., Smyth G.K. (2015). limma powers differential expression analyses for RNA-sequencing and microarray studies. Nucleic Acids Res..

[92] Ashburner M., Ball C.A., Blake J.A., Botstein D., Butler H., Cherry J.M., Davis A.P., Dolinski K., Dwight S.S., Eppig J.T. (2000). Gene ontology: Tool for the unification of biology. Nat. Genet..

[93] Kanehisa M., Goto S. (2000). KEGG: Kyoto Encyclopedia of Genes and Genomes. Nucleic Acids Res..

[94] Croft D., O’Kelly G., Wu G., Haw R., Gillespie M., Matthews L., Caudy M., Garapati P., Gopinath G., Jassal B. (2011). Reactome: A database of reactions, pathways and biological processes. Nucleic Acids Res..

[95] Ru Y., Kechris K.J., Tabakoff B., Hoffman P., Radcliffe R.A., Bowler R., Mahaffey S., Rossi S., Calin G.A., Bemis L. (2014). The multiMiR R package and database: Integration of microRNA–target interactions along with their disease and drug associations. Nucleic Acids Res..

[96] Livak K.J., Schmittgen T.D. (2001). Analysis of relative gene expression data using real-time quantitative PCR and the 2(-Delta Delta C(T)) Method. Methods.

[97] Davis A.P., Wiegers T.C., Johnson R.J., Sciaky D., Wiegers J., Mattingly C.J. (2021). Comparative Toxicogenomics Database (CTD): Update 2021. Nucleic Acids Res..

